# Immune Reconstitution After Allogeneic Haematopoietic Cell Transplantation: From Observational Studies to Targeted Interventions

**DOI:** 10.3389/fped.2021.786017

**Published:** 2022-01-11

**Authors:** Asaf Yanir, Ansgar Schulz, Anita Lawitschka, Stefan Nierkens, Matthias Eyrich

**Affiliations:** ^1^Bone Marrow Transplant Unit, Division of Haematology and Oncology, Schneider Children's Medical Center of Israel, Petach-Tikva, Israel; ^2^The Sackler Faculty of Medicine, Tel-Aviv University, Tel Aviv, Israel; ^3^Department of Pediatrics, University Medical Center Ulm, Ulm, Germany; ^4^St. Anna Children's Hospital, Medical University of Vienna, Vienna, Austria; ^5^St. Anna Children's Cancer Research Institute (CCRI), Vienna, Austria; ^6^Princess Máxima Center for Pediatric Oncology, Utrecht, Netherlands; ^7^Department of Pediatric Hematology, Oncology and Stem Cell Transplantation, University Children's Hospital, University Medical Center, University of Würzburg, Würzburg, Germany

**Keywords:** immune reconstitution, thymic function, peripheral expansion, T-cell receptor repertoire diversity, graft-vs.-host disease, graft-vs.-leukaemia effect, infectious complications

## Abstract

Immune reconstitution (IR) after allogeneic haematopoietic cell transplantation (HCT) represents a central determinant of the clinical post-transplant course, since the majority of transplant-related outcome parameters such as graft-vs.-host disease (GvHD), infectious complications, and relapse are related to the velocity, quantity and quality of immune cell recovery. Younger age at transplant has been identified as the most important positive prognostic factor for favourable IR post-transplant and, indeed, accelerated immune cell recovery in children is most likely the pivotal contributing factor to lower incidences of GvHD and infectious complications in paediatric allogeneic HCT. Although our knowledge about the mechanisms of IR has significantly increased over the recent years, strategies to influence IR are just evolving. In this review, we will discuss different patterns of IR during various time points post-transplant and their impact on outcome. Besides IR patterns and cellular phenotypes, recovery of antigen-specific immune cells, for example virus-specific T cells, has recently gained increasing interest, as certain threshold levels of antigen-specific T cells seem to confer protection against severe viral disease courses. In contrast, the association between IR and a possible graft-vs. leukaemia effect is less well-understood. Finally, we will present current concepts of how to improve IR and how this could change transplant procedures in the near future.

## Introduction

Allogeneic haematopoietic cell transplantation (HCT) establishes a new lymphohaematopoietic system in patients who suffer from severe abnormalities of normal haematopoiesis or immune dysfunction. In the case of malignant disorders of haematopoiesis such as acute lymphoblastic leukaemia (ALL) or acute myeloid leukaemia (AML), the success of HCT critically depends on a graft-vs.-leukaemia (GvL) effect, an immunological reaction in which donor T cells track down and eliminate minimal residual leukaemic cells. HCT creates one of the deepest immunosuppressive states in medicine, sharing many features with naturally occurring states like congenital immune deficiency or human immunodeficiency (HIV) infection. For long it has been known that immune reconstitution (IR) after HCT has to recapitulate immune ontogeny but follows different pathways than nature (1–3). In normal ontogeny, lymphopoiesis begins under protected circumstances *in utero*, is equipped with a perfectly broad repertoire of naïve T cells at delivery, and continues to mature in early childhood when thymic tissue is most active. In contrast, lymphopoiesis post HCT is happening in an aberrant environment, where the thymus is only partially active, organs are damaged from chemotherapy and inflammation, and the body is strongly exposed to internal and external antigens. Furthermore, immune function has to be suppressed around HCT by serotherapy or immunosuppressive drugs to prevent or treat graft-vs.-host disease (GvHD)—an immune-mediated iatrogenic disorder that is caused by the artificial encounter of two immune systems in one organism. Still, the capacity to reconstitute the immune system through the generation and proliferation of immune effector cells is immense (3), and, if guided and supported by targeted interventions, immunity can be restored within months.

**Figure 1:**
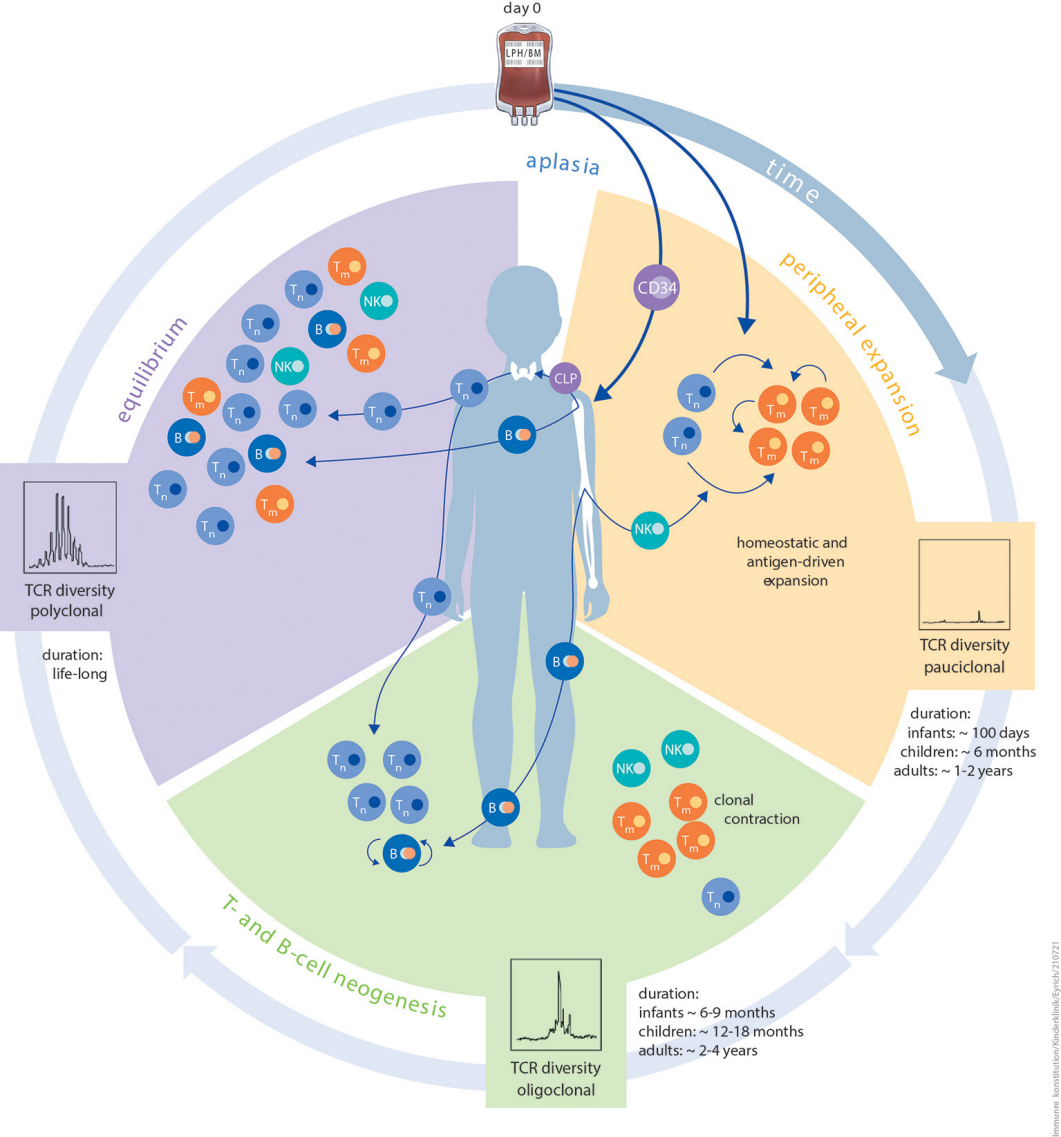
Schematic illustration of the different phases of immune reconstitution following HCT. The first phase *peripheral expansion (orange)* of IR after aplasia is dominated by homeostatic or antigen-driven peripheral expansion of graft-derived T cells. The ratio of naïve T cells to memory T cells is dependent on donor age. The quantity of regenerating T-cell numbers depends on graft size (bone marrow vs. PBSC) and *in vivo* (serotherapy) or *in vitro* T-cell depletion. Diversity of the TCR repertoire during this phase is usually dominated by expansion of singular clonotypes. The duration of this period is strictly influenced by patient age. The second phase *T- and B-cell neogenesis (green)* of IR is characterised by the onset of T- and B-cell neogenesis in the thymus and bone marrow. Thymic and bone marrow niches are more resilient against external stressors and more productive in infants and children than in adults. Other contributing factors are thymic tissue status, application of immunosuppression, and aGvHD or cGvHD. The risk of viral reactivation dramatically reduces as T- and B-cell neogenesis advances. The same probably applies to *de novo* GvHD. In this phase, immunisation with non-live vaccines is feasible. The third and final phase *equilibrium (purple)* of IR is a balanced and stable immune system, which is, to the best of our knowledge, maintained lifelong. Components of innate as well as adaptive immunity reach a level that is relative to patient age. Diversity of the TCR repertoire is polyclonal at this phase. Live, attenuated vaccines can be applied since positive T-cell and B-cell interactions are granted. Autoantibodies tend to disappear and risk of cGvHD is minimal. B, B cell; CLP, common lymphoid progenitor; NK, natural killer cell; TCR, T-cell receptor; T_m_, memory T cell; T_n_, naïve T cell.

IR is a multidimensional process that is unique and variable among different patients (4). It may depend on the graft source, cell dose, human leukocyte antigen (HLA) barriers, conditioning of the patient prior to HCT and post-transplantation interventions, including those to prevent or treat HCT complications. The multitude of variables that influence IR post HCT have been reviewed before (5, 6) and levels of innate and adaptive immune cell reconstitution in transplanted children over time have been reported (7). The transfused graft, in addition to being a source of haematopoietic stem cells for restoration of haematopoiesis, acts as reservoir of immune cells and initiates the complex process of IR. This process is achieved by two different but complementary waves of immune cell regeneration which are closely interlocked and hard to segregate (illustrated in Figure 1). The first wave is mediated by donor lymphocytes present in the graft. Upon transfusion into a lymphodepleted host, these mature lymphocytes have the capability to expand and proliferate in response to antigenic or cytokine-mediated stimulation in a process termed homeostatic peripheral expansion (HPE), providing an early but incomplete immune defence against invading pathogens. More complete IR relies on *de novo* lymphopoiesis from donor-derived stem cells in the bone marrow and/or thymus, a process which can take up to several months.

## Distinct Importance of Naïve and Memory T Cells and Significance of T-Cell Receptor Diversity

Allogeneic HCT grafts include naïve and memory T-cell subsets of which the ratio may differ tremendously between cord blood (mostly naïve cells) and bone marrow or peripheral blood (which have more memory subsets). T memory stem cells (T_SCM_) are of special interest as they show superior reconstitution capacity in preclinical models and contribute to peripheral reconstitution by differentiating into effectors in the early days following haploidentical HCT with post-transplant cyclophosphamide (8, 9). The abundance of naïve T cells in the graft may influence the outcome of patients after allogeneic HCT as long as thymic function has not been restored. Although total numbers of CD4^+^ T cells have been shown to directly correlate with survival post GvHD (10, 11), levels of naïve T cells have been identified as the most potent drivers of alloreactivity (12). In agreement, high levels of CD4^+^ naïve T cells (but not of CD8^+^ T cells) in allografts have been observed to correlate with an increased incidence of acute GvHD (aGvHD) post transplantation (13). These findings led to the initiation of clinical trials using peripheral blood stem cell (PBSC) grafts depleted of naïve T cells, which showed lower rates of aGvHD and chronic GvHD (cGvHD) in HLA-matched HCT, with no apparent increase in relapse rates (14). On the other hand, cord blood grafts (in which almost all T cells are naïve) show great anti-leukaemic potential with reduced relapse risk but a similar likelihood of developing GvHD when compared to bone marrow grafts (15), indicating that T-cell intrinsic factors are contributing to the risk of GvHD development and anti-leukemic efficacy as well. Most significant associations between IR and clinical events were described for CD4^+^ rather than for CD8^+^ T cells, maybe because CD8^+^ T cell numbers fluctuate more swiftly in response to infections (e.g., CMV) or other events post-transplant (16). Still, CD8^+^ T cells numbers have been positively associated with the likelihood to develop GvHD (17, 18), lower relapse rates as well as better overall survival (19). Furthermore, IR of CD8^+^ T cells is highly dependent on the graft type used for transplantation (20): Unmanipulated BM- or PBSC-grafts generally show a more rapid CD8^+^ than CD4^+^ T-cell reconstitution, due to faster homeostatic or antigen-driven expansion of memory-type CD8^+^ T cells. In contrast, after T-cell replete CBT frequently a rapid reappearance of thymus-derived CD4^+^ T cells can be observed (21, 22). However, as IR is influenced by many patient-specific and transplant-related factors, the impact of these patterns on individual outcome is hardly predictable.

Naïve CD4^+^ T cells in particular have been found to undergo HPE and rapidly shift toward a central memory phenotype (22). Although no side-by-side comparisons were made with other graft sources, some authors hypothesised that this CD4^+^ phenotype shift may be a particular characteristic of cord blood T cells (21). In a follow-up study, they showed that the transcription profile of the naïve CD4^+^ T cells from the cord blood grafts overlapped with the profile of foetal CD4^+^ T cells. Likewise, reconstituting cells that were induced in the lymphopenic environment shortly after transplant maintained these overlapping features with foetal CD4^+^ T cells. Interestingly, it was suggested that enhanced T-cell receptor (TCR) signalling *via* the transcription factor AP-1 after ligation of the TCR with self-major histocompatibility complex molecules was responsible for the rapid T-cell reconstitution (23). As expansion of T cells in lymphopenic situations is affected by the strength of TCR activation (24, 25), a skewing toward cells expressing high-affinity TCRs against host and microbiome-associated antigens during HPE may be observed. Thus, beyond monitoring numbers and phenotypes of T cells after HCT, the epigenetic programming and functional status of reconstituting naïve cells should be studies in more detail.

After an age-dependent recovery period in which HPE prevails, the thymus starts to replenish the naïve T-cell pool with new thymic emigrants. Up to this point, the diversity of the TCR repertoire is limited as new TCR recombination events do not take place in donor T cells undergoing HPE. During the first year after T-cell depleted CD34^+^ haploidentical HCT in children, early reconstituting T cells display a predominantly primed, activated phenotype with a severely skewed TCR repertoire (26). Nevertheless, rapidly expanding cells can differentiate into virus-specific T cells that are able to clear an infection within 2 months, as was shown in patients receiving umbilical cord blood (21). Thymopoiesis includes TCR recombination events and positive and negative selection thereby increasing TCR diversity tremendously (27). *Ex vivo* evaluation of thymic function is generally performed by molecular analyses of signal joint TCR excision circles (sjTRECs), which strongly correlate with flow cytometric measurements of recent thymic emigrants (CD45RA^+^CD27^+^CD31^+^ T cells) (28). T-cell diversity analysis can be evaluated by spectratyping the size of the β-chain complementarity determining region 3 (CDR3) (29). Nowadays, next-generation sequencing methods allow high-resolution clonotyping providing quantitative TCR assessments that can be applied to better understand clonotype dynamics during viral infections or GvHD (30) and to identify pathogenic or protective T-cell clones following HCT. In addition, screening the TCR repertoire for absence of sequences with annotated specificity for cytomegalovirus (CMV) (the public CMV repertoire) may also help to identify patients at risk for CMV reactivation and disease who may benefit from prophylactic antiviral strategies (31).

An increase in TCR diversity has been related to a better clinical outcome in multiple studies (30, 32–35). Talvensaari et al. studied TCRs in patients who underwent cord blood transplantation (harbouring an intrinsic, broad, polyclonal TCR repertoire) or bone marrow transplantation (36); they showed abnormal TCR repertoires and low TREC values during the first year after transplantation in both groups. After 2 years, TCR diversity was higher in recipients of cord blood vs. bone marrow HCT (34), suggesting a more efficient thymic regeneration pathway from cord blood lymphoid progenitors despite the lower numbers of CD34^+^ cells in the graft. In turn, recipients of unmanipulated bone marrow from matched sibling donors showed increased TCR diversity and faster T-cell reconstitution compared with children receiving selected CD34^+^ PBSCs from unrelated donors (37). In patients receiving T-cell depleted PBSCs from a matched donor or T-cell depleted haploidentical PBSCs in combination with an independent cord blood product, both GvHD and relapse were independently correlated with lower TCR repertoire diversity (35). In addition, within 6 months, adult cord blood recipients had approximately the same TCR diversity as healthy individuals, whereas recipients of T-cell-depleted PBSC grafts had much lower diversities of CD4^+^ and CD8^+^ T cells. Interestingly, these deficiencies improved 12 months post-transplant for the CD4^+^ but not for CD8^+^ T cell compartment (38). Both TCR repertoire diversity and sjTREC levels can decline during GvHD or infections as a reflection of decreased thymic output under these conditions (39, 40).

Analyses of the diversity of the TCR repertoire were mostly based on the TCR Vβ repertoire in TCRαβ^+^ T cells so far. However, it has been demonstrated that reconstitution of the TCRγδ repertoire is an important marker post HCT as well (41, 42). γ/δ T cells constitute up to ~10% of all T cells in blood; they are effective against virus reactivation and their presence is associated with lower relapse rates after HCT (43, 44). In line with this, Vδ2^neg^ γδ cells isolated from CMV-reactivating patients specifically reacted with both CMV-infected cells as well as leukemic cell lines and primary myeloid leukemic and myeloma cells (45). The interplay between CMV and γδ cell subsets and the result on clinical outcome measures has not been fully elucidated yet (46). In future studies, it would be interesting to assess the predictive value of TCR diversity in specific T-cell subsets with regard to clinical outcomes in more detail, in particular regulatory T (Treg) cells. The latter subset is of special interest as in a murine model adoptively transferred Tregs at the time of HCT accelerated broadening of the TCR Vβ repertoire diversity by preventing GvHD-induced damage in the thymus and secondary lymphoid microenvironment (47).

Given the decisive impact of T-cell IR on survival chances, this issues has to be considered in the design of conditioning regimens. For instance, serotherapy (e.g., with anti-thymocyte globulin; ATG) may reduce the risk of developing GvHD and graft rejection, but dosing should be individualised (based on graft source, absolute lymphocyte count and weight) to prevent dramatically reduced T-cell IR in patients after high ATG exposure (48–51), in particular when given in combination with filgrastim (52).

## Differences in Immune Reconstitution Between adults and Children

Factors affecting IR have been actively investigated for almost 30 years now. Besides other contributing factors such as stem cell dose (53), donor age (54, 55), and mixed chimerism (56), patient age at transplantation has been recognised as a prime determinant of the speed and quality of IR from the start of this research (57). The T-cell compartment (both CD4^+^ and CD8^+^) reconstitutes slower in adults than in children, which translates into a higher rate of life-threatening opportunistic infections in older patients. Storek et al. already reported in 1995 that T-cell phenotypes in adult HCT recipients were strikingly different from neonatal T cells and that these changes were more pronounced in the CD4^+^ compartment (58). Numerous later studies confirmed this finding and supported the notion that the second, thymus-dependent wave of T-cell reconstitution is enhanced in children (59). Prediction models of thymic output based on TREC measurements revealed that thymic reconstitution can start as early as 83 days post-transplant in infants and that each additional year of patient age adds 2 weeks to that starting point (60). Interestingly, this advantage of children with regard to improved naïve T-cell regeneration seems to confer protection against viral infections (57), non-relapse mortality and cGvHD (61) but not aGvHD and leukemic relapse, because relapse incidences in paediatric and adult ALL patients after allogeneic HCT are not strikingly different (62, 63). Whether the increased thymic output contributes to a better GvL effect is unknown. However, regeneration of functional Tregs, probably derived from thymic Treg precursors, is a prerequisite for resolution of cGvHD in children (64). Therefore, it is conceivable that the addition of new, potentially leukaemia-reactive clonotypes to the TCR repertoire is counterbalanced by the regeneration of tolerizing Tregs. Mechanistic studies addressing this issue are lacking so far. Furthermore, the precise mechanisms underlying improved thymic reconstitution (increased thymic cellularity, higher susceptibility of thymic precursors to cytokines, or enhanced influx of committed lymphoid progenitors) have not been elucidated so far. Nevertheless, enhancing thymic reconstitution in adults to achieve the same level as that observed in children is a pivotal strategy to boost IR (see below).

Another, less-examined difference between children and adults may be the better preservation of the B-cell bone marrow niche in children. Children show faster reconstitution of total numbers of B cells (56), have more B-cell precursors in regenerating bone marrow (65), and exhibit more B-cell neogenesis as measured by kappa-deleting recombination excision circles than do adults (66). Moreover, cGvHD has been demonstrated to have little impact on B-cell neogenesis and bone marrow precursor composition in children (67), which is in stark contrast to observations in adults (68, 69). Therefore, the microenvironment of the thymus as well as the bone marrow seems to be more resilient to noxious influences such as conditioning regimens and alloreactivity in children compared to in adults. These complex interactions (e.g., regenerating CD4^+^ T cells providing help to transitional and naïve B cells), contribute to facilitate new humoral immune responses (56) and lower production of autoantibodies (67).

In contrast to the aberrant pathways of adaptive immunity regeneration after allogeneic HCT, natural killer (NK)-cell reconstitution after HCT seems to resemble NK-cell ontogeny in early childhood, with a preponderance of immature NK cells in the early post-transplant phase (70). Type of graft manipulation (NK-replete vs. NK-depleted grafts) seems to have a greater impact on NK reconstitution than patient age, although no comparative studies directly addressing this question are available. In a heterogenous cohort of paediatric ALL patients who underwent haploidentical HCT, T-cell depletion techniques that also depleted graft-derived NK cells (i.e., CD34^+^ selection) resulted in faster NK-cell recovery post-transplant than techniques like CD3/CD19-depletion, which keep NK cells in the graft (71), underlining the importance of cytokine sinks such as interleukin (IL)-7 and−15 for NK-cell development (72). Especially in the haploidentical transplant setting, potential NK cell alloreactivity has gained a lot of attention. Differences in the killer-cell immunoglobuline-like (KIR) gene haplotype could lead to a donor NK cell activation caused by the lack of an inhibitory receptor on host leukemic cells. The clinical relevance of this scenario remains controversial. One study analysing 85 children with ALL transplanted with *ex vivo* T-cell depleted haploidentical PBSCs showed a benefit if the donor had a KIR B content score (5-year event-free survival of 51 vs. 30% in KIR B vs. KIR A haplotype, respectively) (73). However, this was not confirmed in a subsequent study of 80 children with acute leukaemias receiving TCRab/CD19-depleted haplo grafts. Here, KIR-KIR-L mismatching was not associated with any difference in leukaemia-free survival (74). For more details on that issue we refer to one of the excellent reviews published recently (75).

## Immune Reconstitution and Viruses

### The Complex Relationship Between Antigen Exposure and Immune Reconstitution

Exposure to infectious agents in the early post-HCT period puts the patient at increased risk for morbidity and also alters the process of IR, increasing risks of further infections and immune-mediated diseases. In order to prevent such exposure, patients are usually instructed to keep socially distanced or isolate from others, restrict their diet and take other behavioural measures in the post-HCT period to minimise their risk of encountering exogenous infections. However, as patients have already encountered infections prior to HCT, any viruses that remain latent in their body (and that are usually under tight control of the normal immune system) might become reactivated post HCT and cause significant morbidity and mortality. The best studied viral reactivation post allogeneic HCT is CMV reactivation; however, other viruses such as Epstein-Barr virus (EBV), human herpesvirus 6 (HHV-6), adenovirus and BK polyomavirus are also of clinical importance. Each virus causes a distinct pattern of disease and can appear at different levels of immunosuppression (76).

#### Cytomegalovirus

CMV has been considered for many decades to be the leading cause of infectious complications in recipients of bone marrow transplants (77) and, as such, serves as the prototype for the study of viral reactivation and IR post HCT. Since CMV is ubiquitous worldwide, infection usually occurs in childhood and most patients are seropositive at the time of HCT. The standard of care is to monitor CMV levels by weekly polymerase chain reaction (PCR) testing and to treat any emerging reactivation pre-emptively before clinical disease emerges. Many studies have investigated the kinetics, risk factors and clinical outcome of CMV reactivation (78). Seropositive recipients receiving a graft from a seronegative donor are at highest risk for CMV reactivation (79), reflecting the central role of specific memory T cells from the graft in controlling CMV reactivation in the early post-HCT period. Aubert et al. have shown that healthy seropositive individuals have a significant percentage (median 1.3%; range 0.29–5%) of memory CD8^+^ cells which are specific for the E42 epitope of the CMV pp65 protein, and that these cells are capable of mediating immune protection against CMV (80). In the context of HCT, a clear inverse correlation was found between low numbers of these cells and CMV reactivation. Interestingly, following viral reactivation, the number of E42-epitope-positive CD8^+^ cells increased dramatically, reflecting the ability of these cells to proliferate and expand in response to antigenic stimuli regardless of the presence of CD4^+^ helper cells, resulting in viral clearance.

The presence of these memory CD8^+^ cells immediately after HCT varies among individuals according to graft composition and the degree of T-cell depletion. In a large series published recently (81), the authors showed that patients with high peak CMV titres (>20,000 copies/mL) had significantly lower numbers of T cells (both CD4^+^ and CD8^+^) at both 1 and 3 months post HCT but these numbers increased later on, becoming high at around 6 months. Interestingly, patients who did not have reactivation of CMV (<500 copies/mL) did not show this elevation in T cells and had significantly lower numbers of T cells at 1 year post HCT. These findings are in accordance with another trial studying general IR patterns post HCT using 25 lymphocytes subsets (82). Using multivariate methods, those researchers showed that CMV reactivation and cGvHD are the major determinants of IR patterns at 1 year post HCT. Lymphocyte subsets from seropositive patients clustered differently to those from CMV seronegative patients, with increased proportions of activated, late memory effector CD8^+^ T cells and reduced B-cell subsets observed in seropositive patients. Due to the persistence of CMV antigens during viral latency, the long-term memory T-cell pool accumulates T cells with CMV specificity, a phenomenon called memory inflation.

Furthermore, few studies have demonstrated a bidirectional relationship between CMV reactivation and the occurrence of GvHD. While the observation that CMV reactivation is a consequence of GvHD treatment is intuitively understandable, these studies have demonstrated the converse, showing increased occurrence of GvHD following CMV reactivation (83, 84). Few hypotheses regarding this etiological relationship have been tested including induction of HLA class II expression following CMV reactivation (85), or sequence homology between CMV and human tissue peptides (86). Regardless of the biological explanation, this association as well as the above-mentioned studies regarding the impact of CMV reactivation on T-cell subpopulations, highlight the importance of CMV reactivation post HCT not only as the leading infectious agent but also as a key player in shaping the IR post HCT.

The recent introduction of Letermovir as a very efficient agent in preventing CMV reactivation post HCT, allowed us for the first time to assess IR patterns in the absence of CMV reactivation. Several groups have collected data regarding this question: Sperotto et al. have shown that patients who received prophylactic letermovir, had significantly lower CD4 and CD8 counts at 2 and 3 months post HCT, compared to patients who were treated by a standard preemptive approach (87). From a functional perspective, Zamora et al. have recently demonstrated that patients who received letermovir have significant lower levels of functional CMV-specific T cells (88). Albeit further data is definitely needed, these studies again emphasise the crucial role of CMV reactivation in shaping IR patterns post HCT.

#### Epstein-Barr Virus

In contrast to CMV, EBV reactivation post HCT originates usually from graft-derived donor B cells that under strong immunosuppression loose the tight control of EBV-specific T cells, resulting in a spectrum of disorders called post-transplant lymphoproliferative disease (PTLD). EBV reactivation is less common than CMV, tends to appear slightly later after HCT, and seems to require a deeper immune suppression (89). Standards for diagnosis of PTLD and treatment of EBV reactivation are less stringent than that for CMV as there is no consensus on the level of EBV copy numbers that puts the patient at high risk for PTLD. Since EBV is not targetable by antiviral drugs, a CD20 mAbs and EBV-specific T-cells remain the only available treatments so far.

D'aveni et al. profiled the immune response to EBV using the ELISpot assay at 60, 100, 180, and 360 days post HCT in 28 patients transplanted for both malignant and non-malignant indications (90). Not surprisingly, they found a correlation between general T-cell reconstitution and EBV-specific reconstitution, as well as significantly earlier and higher reconstitution in paediatric vs. adult patients. In this small series, patients with an ELISpot result of more than 1,000 spot-forming cells (SFC)/10^6^ mononuclear cells still had the ability to clear the virus spontaneously without treatment. Similarly to the picture with CMV immunity, EBV antigenic stimulation was the strongest driver of proliferation of these cells, but this effect disappeared 1 year post HCT, suggesting that, unlike CMV, EBV reactivation has no effect on long-term IR. In a relatively large series published by Stocker et al., treatment of EBV reactivation with anti-CD20 monoclonal antibodies did not result in a different IR pattern than that observed in patients without anti CD20 treatment with the exception of delayed B-cell recovery, which normalised after 1 year post HCT (91). This delayed B-cell recovery was mirrored clinically by a higher need for immunoglobulin (Ig) G replacement in the anti-CD20 group than in the non-anti-CD20 group. Frequency of infections and clinical outcome did not differ between treatment groups.

#### Adenovirus

Adenovirus reactivations are of particular interest in the paediatric population (92). As no highly effective antiviral treatment against adenovirus exists, reactivation has emerged in the recent years as a major cause of morbidity and mortality after HCT in children. Admiraal et al. found that CD4^+^ T-cell reconstitution was the only immunological predictor of adenovirus reactivation (16). The chance of reactivation was reduced by 5% with every 10 cells/μL increase in CD4^+^ T cells. Furthermore, patients with early CD4^+^ T cell reconstitution (defined as CD4^+^ T cells >50 cells/μL in two consecutive samples before day +100) had a shorter duration of viraemia and, on survival analysis, had the same favourable outcome as patients without adenovirus reactivation. This is in contrast to the dismal prognosis observed in patients with adenovirus reactivation without CD4^+^ T-cell reconstitution.

### Human Herpesvirus 6

HHV-6 is the most common virus to reactivate post HCT, but cases with clinical disease (i.e., encephalitis) are rare (93). De Koning et al. found that the only predictor of HHV-6 reactivation was CD4^+^ IR (94). Interestingly, HHV-6 reactivation was found to be a strong predictor of grade II–IV GvHD, and this effect vanished if CD4^+^ IR had occurred. Furthermore, in subsequent work, HHV-6 had a significantly negative impact on numbers of CD4^+^ T cells 1 year post HCT, possibly caused by the cytopathic effect of HHV-6 on thymopoiesis (95). This effect was reversed if antivirals were used.

### Other Viruses

Other viral reactivations (e.g., BK polyomavirus, varicella zoster virus, and herpes simplex virus) have been less studied systematically in terms of IR, but case reports point toward a central role of T-cell immunity in controlling these reactivations following HCT (76).

### Section Conclusion

To conclude this section, viral reactivations mirror the status of T-cell reconstitution. CD8^+^ memory T-cell populations seem to mediate protection against or clearance of CMV and EBV, whereas for other Herpesviridae such as adenovirus or HHV-6, CD4^+^ T cell counts are the main predictor for both reactivation and outcome. CD4^+^ T-cell counts are also the main predictor for long-term anti-CMV immunity. CMV reactivation is a strong stimulator of global T-cell reconstitution, with the highest effect observed 6 months post HCT. HHV-6 reactivation might have the opposite effect, with patients who experience reactivation tending to have lower T-cell counts at 6 months and 1 year post HCT. Adoptive transfer of antigen-specific T cells will probably gain widespread use in the near future, as this therapy directly targets the mechanisms behind viral reactivation.

## Immune Reconstitution and Acute GVHD: The “Chicken and Egg” Dilemma

GvHD is a frequent complication of HCT. Although the incidence is lower in paediatric compared to in adult patients, GvHD significantly contributes to transplant-associated morbidity and mortality. It is broadly accepted that aGvHD and cGvHD involve different effectors and targets and have different pathologic pathways, therefore being seen as two different diseases. Nevertheless, aGVHD remains the major risk factor for development of cGvHD in the paediatric population (96, 97). This review focuses on parameters and kinetics of early IR of mainly the adaptive immune system, and therefore this chapter will cover primarily aGVHD.

In general, aGVHD is mediated by alloreactive donor T cells activated by host antigen-presenting cells followed by donor cell reactivity against a variety of target tissues of the host. aGVHD is associated with significantly impaired IR, but which is the cause and which is the effect? This question applies to a number of interacting aspects: (1) the T- and the B-cell compartment, as the antigen-presenting cells involved in aGvHD could be B cells; (2) the number and function of subpopulations of the adaptive immune system (quantity and quality); (3) HPE vs. impaired thymic production; (4) the composition of the graft and the microenvironment of the host; and (5) the effects of aGvHD itself and the administration of immunosuppressive agents for GvHD prophylaxis and treatment.

Immune cell function does not equate to cell number: it is important to distinguish between quantitative immune cell reconstitution and qualitative IR. For instance, T cells often remain dysfunctional after HCT, with a skewed TCR repertoire even after recovery to normal number (98). Hence, the normalisation of B- and T-cell numbers does not necessarily indicate reconstitution of their function and it has been suggested to differentiate between “immune reconstitution” and “immune recovery” rather than using IR alone (99).

Data regarding the influence of aGVHD on IR profiles and vice versa lack detailed information on reconstituting cell subsets and on effector functionality. Moreover, as IR is age dependent, this and other reviews are hampered by the lack of data from a primarily paediatric setting (7). Table 1 provides published data on immune cell subsets in adaptive IR and their relation to aGvHD after HCT in paediatric and adult patients (10, 11, 17–19, 49, 98, 100–110).

**Table 1:** Immune reconstitution parameters and reported association with acute GvHD.

**Immune cell subset**	**Interaction with acute GVH**	**Age group**	**References**	**Comments**
CD4^+^ Th cells	Higher numbers attenuate aGvHD	Paediatric	(98)	Often, CD4^+^ T cells not only include Th but also Treg (98)
	CD4^+^ IR had no impact on aGvHD		(49)	
	Increased CD4^+^ at day +28 associated with increased risk of aGvHD	Paediatric/adolescent	(100)	
	Early CD4^+^ IR predictive for better outcome after aGvHD	Paediatric	(10)	
	No impact of CD4+ IR on aGvHD		(11)	
TREC level	High sjTREC levels correlate with lower incidence of aGvHD grade II–IV	Adult/adolescent	(101)	Ratio of sjTREC to βTREC may mark thymic proliferation
	Sj and βTRECs levels lower in aGvHD at >6 months			
	Recovery of thymic output in resolved aGvHD at >12 months in adolescents (<25 years old)		(102)	
CD8^+^ T cells	Early recovery associated with increased risk of aGvHD	Adult	(100)	
	Increased CD4^+^ T cells at day +28 associated with increased risk of aGvHD			
	High numbers of T_EM_ (CD38^bright^CD8^+^ effector memory T cells) predict aGvHD	Paediatric/adult	(17)	
	Increase of T_EM_ in median 8 days before aGvHD onset			
CD4^+^ T_reg_ cells	Higher numbers associated with less aGvHD	Paediatric/adult	(98)	T_regs_ can be subdivided into naturally occurring and induced cells
	Inverse correlation between T_reg_ numbers and grade of aGvHD	Paediatric/adolescent	(103)	
	Low CD4^+^FoxP3 T_regs_ at day +30 are associated with increased risk of grade II–IV aGvHD		(104)	
B cells	Early recovery associated with decreased risk of aGvHD	Paediatric	(105)	Most paediatric data on B-cell IR and aGVHD cover CD19+ cells only (5). Most studies on B-cell IR and aGVHD report on cGVHD (105)
	Low numbers of B cells and naïve B cells at day +56 associated with increased risk of grade II–IV aGvHD	Adult	(100)	
	Lower B cells numbers in patients with a history of grade II–IV aGvHD		(18)	
iNKT cells	Early recovery associated with lower risk of aGvHD	Paediatric	(105)	
		Paediatric/adult	(98)	
	Lower levels independent risk factor for aGvHD	Adult	(106)	
γδ T cells	No association with aGvHD	Paediatric/adult	(107)	
	Lower numbers of γδ T cells associated with history of grade II–IV aGvHD	Adult	(18)	
	Lower numbers of γδ T cells in aGvHD		(108)	
	Risk of aGvHD lower with higher numbers of γδ T cells at day +28		(19)	
MAIT cells	Low numbers are a risk factor for aGVHD	Paediatric/adult	(109)	
	Lower MAIT cell counts (peripheral blood) in aGVHD	Adult	(110)	
	*In vitro* MAIT cells suppress T cell proliferation, which may impact aGVHD			

*aGvHD, acute graft-vs.-host disease; iNKT, invariant natural killer T; IR, immune reconstitution; MAIT, mucosal associated variant T; sjTREC, signal joint T-cell receptor rearrangement excision; TEM, T effector memory; Th, T helper cell; TREC, T-cell receptor excision circle*.

### CD4^+^ T Cells

Perturbations of both HPE and thymic output contribute to impaired CD4^+^ T-cell reconstitution in patients with aGVHD. In this regard, patient and transplant associated aspects such age, sex, underlying disease, genetic differences between donor and host, stem cell source, and type of conditioning are influencing factors for the IR of CD4^+^ T cells.

In general, aGvHD is characterised by the predominance of effector CD4^+^ cells that are capable of secreting inflammatory cytokines and that mediate tissue damage (111). Additionally, in aGVHD allo-reactive T cells directly target both the lymphoid and the epithelial components of thymic architecture. Allo-reactive T cells further limit renewal of thymic cellularity after conditioning therapy, thereby preventing negative selection of alloreactive T cells which subsequently promote GvHD (112). Thus, IR is stuck in a vicious circle of arrested thymus regeneration and impaired *de novo* production of diverse T cells (113). This results in the compromised production of naïve T cells together with a shortened survival and higher susceptibility to apoptotic cell death of T cells due to the overexpression of death receptors and the under-expression of pro-survival proteins (114–116). This is accompanied by a reduced production of cytokines indispensible for thymopoiesis, which in turn leads to lower TREC levels and a distorted TCR repertoire (40, 117–119).

In pre-clinical models, it has been demonstrated that T cells from animals with GvHD were capable of significant expansion, molecular diversity and repertoire regeneration after their transfer into secondary hosts, indicating that deficits in the T-cell repertoire are not necessarily fixed but may have the capacity for normalisation once they are removed from the GvHD milieu. Therefore, the GvHD microenvironment of the host seems to be responsible for quantitative and qualitative failure of effective CD4^+^ T-cell reconstitution during GvHD (111, 118, 119).

In clinical studies, aGvHD correlates with aggravated skewing of the TCR repertoires of both CD4^+^ and CD8^+^ T cells as well as antigen-specific T cells. Both T- and B-cell lymphopenia and an inadequate repertoire of CD4^+^ and CD8^+^ T cells for at least 1 year after transplant increase the risk of recurrent reactivation of latent viruses, which may further contribute to a higher risk for development of aGvHD (120).

Koning et al. reported a retrospective dual-centre study of CD4^+^ T-cell reconstitution in paediatric patients following HCT with an aim to identify predictors of survival outcomes after aGvHD (10). Achieving CD4^+^ T-cell IR within 100 days after HCT did not decrease the risk of developing aGvHD but was strongly predictive for better survival outcomes (non-relapse mortality and overall survival) after moderate-to-severe aGvHD. Generally, conventional HCT grafts are associated with a higher proportion and an earlier recovery of Tregs together with greater TCR diversity when compared with T-cell-depleted grafts. Of note, de Koning et al. reported that for both cohorts (the conventional HCT and the T-cell-depleted HCT group), early CD4^+^ T-cell IR correlated significantly with better outcomes of aGvHD.

By means of sjTREC and beta-T-cell receptor excision circles (βTREC) quantifications, a significant but transient reduction in thymic output as well as in early thymocyte differentiation in patients with aGvHD was shown by Clave et al. in a cohort including adolescent patients after matched sibling donor HCT performed mainly for malignancies (101). Interestingly, in these patients who were <25 years old, thymic function recovered at 1 year, indicating that the impact of aGvHD on the adolescent thymus could be transient. Gabella et al. confirmed that sjTREC levels were not affected by aGvHD during long-term follow-up of adult and paediatric patients after HCT in mainly malignant diseases with myeloablative conditioning (102).

The association between cGvHD and low TREC levels indicative of poor thymic function was described by Olkinuora et al. in a prospective paediatric study: in this cohort, low TREC levels correlated with high mortality rates (121).

### CD8^+^ T Cells

Early donor T-cell expansion is characterised by mainly CD8^+^ cells with a restricted repertoire and of memory cell type. The IR pattern of CD8^+^ T cells differs to that of CD4^+^ T cells, e.g., in that expanded CD8^+^CD28^−^ effector memory T cells can dominate for more than 2 years post HCT (111). Expanded oligoclonal CD8^+^ cells are associated with an increased risk of aGvHD (18, 122, 123).

### Regulatory T Cells

T_regs_ (CD4^+^25^+^FoxP3^+^) are known to maintain immune homeostasis and tolerance by inhibiting cytokine secretion and proliferation of various effector cells. They can be subdivided into naturally thymus-derived T_regs_ and induced Tregs differentiated from non-regulatory CD4^+^25^+^ cells. Adoptive transfer of *ex vivo* expanded T_regs_ has been shown to result in superior immune reconstitution and less GvHD in preclinical murine allotransplant models (124). Full T_reg_ reconstitution prevents the rapid oligoclonal proliferation that gives rise to pathogenic CD4^+^ effector T cells, while preserving the slow homeostatic form of lymphopenia-induced peripheral expansion that repopulates a diverse peripheral T-cell pool (125). This effect is mediated through CTLA4-dependent downregulation of CD80 and CD86 on dendritic cells by T_regs_.

Regarding clinical data, an association between T_reg_ numbers and incidence of aGvHD has been established: A higher T_reg_ content in the graft confers lower non-relapse mortality and improved overall survival (126). Magenau et al. reported that in adult and paediatric patients with aGvHD after a matched sibling or matched unrelated donor HCT, T_reg_ frequencies were inversely correlated with aGvHD grading. T_reg_ frequencies were measured at disease onset as the percentage of CD4^+^CD25^bright^Foxp3^+^ T cells out of total nucleated cells (103). Rezvani et al. were able to show that a low CD4^+^FOXP3^+^ T-cell count early after HCT (day +30) was associated with an increased risk of grade II–IV aGvHD in adult and adolescent patients who underwent HCT (104). Clinical trials with adoptive transfer of T_reg_ are described in more detail in section Cellular Therapies. Cellular Therapies (see below).

Noteworthy, T_regs_ may also play a role in the graft-vs.-leukaemia reaction. In a series of 85 patients with leukemic relapses after HCT, a higher content of Helios^+^ T_reg_ at day +30 within the CD4 compartment was accompanied by a higher incidence and earlier occurrence of leukemic relapse (127). In contrast, checkpoint blockade which is applied to increase antitumor immunity both in the autologous and the allogeneic setting is known to inhibit T_regs_. Patients with advanced/metastatic solid tumours receiving aPD-1 and aCCR4 checkpoint inhibitor infusions had a reduced effector T_reg_ population (128). Patients who received aCTLA4 infusions for the treatment of leukemic relapses after allo HCT showed diminished counts and less activated T_regs_ but exhibited a 35% likelihood of developing immune-related adverse events or GvHD. These data show that T_regs_ are key players in the regulation of autoimmunity and may tip the balance between GvH and GvL.

### B Cells

The first B cells to emerge into the periphery following HCT are CD19^+^CD21^low^CD38^high^ transitional B cells; the percentage of these cells subsequently decreases while mature CD19^+^CD21^high^CD27^neg^ naïve B cells are replenished (120). However, most paediatric studies provide information on CD19^+^ B cells alone (7). Generally, GvHD is correlated with impaired IR of the B-cell compartment, with regards to both numbers and function, yet most reported data are in the context of cGvHD (105). Abdel-Azim et al. observed in paediatric HCT recipients the normalisation of numbers of naïve B cells by 6 months together with a deficiency of IgM^+^ memory B cells and switched memory B cells (129). While the latter normalised within the first year after HCT, the deficiency of IgM memory B cells persisted for up to 2 years. They concluded that paediatric HCT recipients have impaired humoral IR, predominantly owing to a blockade of IgM memory B-cell maturation compared with earlier T cell-dependent switched memory cell IR.

### Profiles of Immune Reconstitution Associated With Acute GvHD

Bae et al. reported no significant impact of aGvHD on lymphoid IR in paediatric patients who underwent HCT for malignant diseases (20). In recent research by Schultz et al. evaluating immune profiles at day +100 after HCT in correlation with National Institutes for Health (NIH)-defined GvHD, the authors described distorted patterns of IR after resolved aGvHD and late aGvHD at day +100. They then compared theses immune profiles to an immunological fingerprint of patients without any history of GvHD (immune-tolerant patients). They identified a number of different associations per group and found a progression of immune abnormalities from no cGvHD to late aGvHD, and further to the most complex pattern in cGvHD (130).

Models of immune function have been published that aim to reflect various subpopulations of immune cells and also to consider different patterns of IR (70, 131). A three-component multivariate model with a reference domain of ellipsoidal shape based on normal leukocyte subtype values from healthy children and adolescents has been created by Koenig et al. This model was used to classify paediatric patients as having high or low risk for a post-HCT events based on their IR status; significantly higher number of HCT survivors mainly after malignant diseases and various conditioning regimens fell into the low-risk vs. high-risk group during follow-up (day +200 and day +300) (132). Mellgren et al. used a principal component analysis to better analyse the process of IR after paediatric HCT. They were able to show that dysfunctional IR patterns precede severe complications such as cGvHD, relapse, and death (133). Although these reports do not provide conclusive data regarding the interaction of aGvHD and IR, they aid understanding of the interactions between variables after HCT and support a more differentiated and meaningful viewpoint on IR and transplant-related complications such as GvHD.

### Haematopoietic Niche and Acute GVHD

von Bonin et al. outlined in a comprehensive review that both haematopoietic cells and cells forming the haematopoietic/progenitor niche of the bone marrow have been identified as targets in GvHD. Haematopoiesis in general and B-cell neogenesis in particular are affected by the toxic environment of GvHD, leading to a shift toward myelopoiesis (134). In terms of the *in vitro* composition and function of the haematopoietic microenvironment, Martinez-Jaramillo et al. found decreased numbers of myeloid, erythroid and multipotent progenitor cells in recipients of bone marrow transplants in comparison with healthy controls. Of note, progenitor levels were significantly lower in patients with GvHD (7% of normal marrow levels in patients with GvHD vs. 44% of normal marrow levels in patients without GvHD). These findings corresponded with the severely reduced numbers of fibroblastic progenitors and adherent stromal cells observed in long-term marrow culture in patients with GvHD vs. those without (135).

## Immune Reconstitution and Graft-vs.-Leukaemia Effect

Immune attack of donor T cells against residual host leukaemic cells is a major pathway by which allogeneic HCT combats haematological malignancies. In general, a higher number of T cells in the graft is associated with lower relapse rates but at the cost of a higher incidence of GvHD (136). Patients with early recovery of antiviral T-cell responses have a higher probability of relapse-free survival (137), and high numbers of interferon gamma (IFNg)-reactive T cells during early IR have been shown to be associated with improved overall survival (138). However, certainly not all donor T cells contribute to the supposed GvL effect and the involved specific T-cell subpopulations are not known so far.

Since the first reports of the contribution of an immunological GvL effect on the success of HCT in the 1980s (139), the segregation of the GvL effect from GvHD has been considered the “holy grail” of HCT. In the quest to enhance the GvL effect without increasing the risk for GvHD, two general approaches have been studied. The first approach aims to discriminate subpopulations of T cells that can mediate GvL from those that mediate GvHD, thereby enabling a safer and more effective T-cell composition by graft engineering. The second approach tries to define minor histocompatibility antigens that are restricted to the haematopoietic lineage and to elicit specific T-cell responses against these antigens post HCT. Though both approaches have not yet been translated into clinical routine, progress has been achieved and some modalities are currently tested in clinical trials.

Zheng et al. have shown in a murine model of chronic myeloid leukaemia that donor memory CD4^+^ T cells (CD4^+^CD62L^−^CD44^+^CD25^−^) can kill leukaemic cells without causing GvHD, as opposed to the action of naïve cells that cause GvHD (140). The authors speculated that the reason for this difference is that memory T cells can generate only a limited immune response that is sufficient for GvL but not sufficient to cause GvHD (which requires a high-magnitude response and high systemic levels of cytokines to cause tissue invasion and systemic inflammation). The same group has also shown that adoptive transfer of CD8^+^ memory T cells from donors vaccinated against the recipient minor histocompatibility antigen H60 augmented the GvL effect without increasing GvHD (141).

Given these pre-clinical data about the central role of memory T cells in GvL, Triplett et al., conducted a clinical trial (142) in 17 paediatric patients with relapsed/refractory acute leukaemia, performing reduced intensity HCT from haploidentical donors after naïve (CD45RA^+^) T-cell depletion of the graft. At a median follow-up of 223 days, aGvHD rate was acceptable, and there were only two cases of relapse: both of these were in patients with advanced AML in whom primary induction had failed. Interestingly, nine patients had detectable disease at time of transplant, yet relapse rates were still low, highlighting the potential of memory T cells to mediate GvL effect. A second trial using transfer of CD45RA-depleted T cells in 35 patients with high-risk acute leukaemia confirmed low rates of cGvHD (9% with a follow-up of 932 days). aGvHD rates were similar to T-replete HCTs (66%; 95% CI 41–74%) but all cases of aGvHD were steroid responsive and no patient required second line treatment. Overall survival was 78% at 2 years, which is encouraging in this high-risk population (14). These data suggest that CD45RA^−^ memory T cells are not devoid of any GvHD potential; however, GvHD seems more controllable for this type of HCT. The combination of CD45RA with other surface antigens such as CD276 as a depletion marker can confer superior protection against GvHD initiation (143).

Potential targets for donor T cells in the HCT setting are any polymorphic proteins of the host against which donor T cells have not been tolerized during their education in the host thymus. Recent molecular analyses have revealed that 12% of the human exome is polymorphic but only 0.5% of all single nucleotide variants (SNVs) are finally presented as HLA class I peptides (144). From this huge number of possible antigens, about 50 candidates have been biologically validated as *bona fide* minor histocompatibility antigens with relevance for HCT (145). Early mechanistic studies revealed that T-cell responses against minor histocompatibility antigens are oligoclonal in nature and CD4^+^ dominated (146, 147); one or a few minor histocompatibility antigen mismatches can be sufficient to cause GvHD and massive thymic infiltration (148). Disappointingly, a closer look at donor–host minor histocompatibility antigen disparities has not allowed clear separation between GvL and GvHD so far. The *UGT2B17* truncating gene deletion has been shown to lead to increased incidence of aGvHD and reduced survival in HCT recipients (149), while HA-8 and ACC-1 SNVs in the recipient have been associated with an increased incidence of cGvHD (150). In a large retrospective analysis, Spierings et al. (151) investigated in 849 HLA-matched HCTs the impact of 10 autosomal and 10 HY-encoded minor histocompatibility antigens on GvHD and relapse incidence. Their most striking observation was a lower relapse rate and higher overall survival in patients mismatched for haematopoiesis-restricted minor histocompatibility antigens compared to patients who were matched in these antigens. Notably, this association was only given in the context of GvHD (not without).

The introduction of immune checkpoint inhibitors as an efficient method of immune-based anti-cancer therapy made its use in the context of allo HCT an intriguing way to augment the GvL effect. Pilot reports about patients with Hodgkin disease who relapsed post allo HCT have shown that this modality can be effective, though carrying the risk of occurrence of *de novo* GVHD (152). Davids et al. have prospectively treated 28 adult patients with relapsed haematological malignancies post HCT with aCTLA4 blockade and other 28 adult patients with aPD-1 blockade (153, 154). While some responses were noted (more in lymphoid diseases and some complete responses in extramedullary myeloid leukaemia), severe GvHD and other serious immune-mediated adverse events occurred in a significant proportion of patients. Of note only a single ALL patient was included in these studies. Further and more homogenous studies are required to better characterise patients in whom the potential benefit of immune checkpoint blockade overrides its risks. Noteworthy, in a study of 85 patients after allo HCT for various haematologic malignancies, LAG-3 and TIM-3 rather than PD-1 were overexpressed on T-cells of relapsing patients, indicating that other exhaustion markers beyond the PD-1-PD-L1 axis might be interesting and druggable targets to enhance GvL after allo HCT (127).

In summary, these data indicate that natural IR will most likely not distinguish between GvHD and GvL effects. However, adoptive transfer of minor-antigen-directed T cells, the generation of which is challenging but feasible (155, 156), in a T-cell depleted setting should be the subject of further research. Another approach to skew IR toward preferred regeneration of minor-antigen-specific T cells is the vaccination of the recipient with minor-peptide-loaded dendritic cells in combination with donor lymphocyte infusions (DLIs) (157). Given the very tight association between GvL and GvHD, a clearer separation of these two effects will only be possible by controlling IR through tailored grafts and targeted add-back of TCR specificities, e.g., antiviral T cells in the first 2–3 months to avoid or control viral reactivations followed by adoptive transfer of donor T cells reactive against leukaemic epitopes.

### The Problem of Slow Immune Reconstitution

As outlined above, delayed IR—and in particular T-cell reconstitution—is associated with clinical complications following HCT. The delay of IR may be the reason or the result of these complications—probably the interaction works both ways in most instances. To optimise the outcome of HCT, slow IR should be prevented or treated. This can be performed either by avoiding factors that impede reconstitution or by using procedures that improve the reconstitution.

#### Avoiding Factors That Impede Immune Reconstitution

Serotherapy, total body irradiation and prophylactic immunosuppression are known inhibitors of prompt IR; however, they are indispensable elements of many conditioning regimens. Viral reactivations can impede or skew IR, as extensively discussed above. Prophylactic or pre-emptive strategies aim at avoiding viral reactivations and disease. Also mentioned above is the impact of aGvHD on IR. Prevention and treatment of aGvHD should focus on methods (e.g., selective allodepletion or extracorpeal photopheresis) that preserve T-cell function against viruses or other non-GvHD targets. Because avoidance of these detrimental factors is not always possible in clinical practise, substantial efforts have been undertaken to establish new techniques for improvement of IR (see below).

#### Procedures That Improve Immune Reconstitution

**Figure 2:**
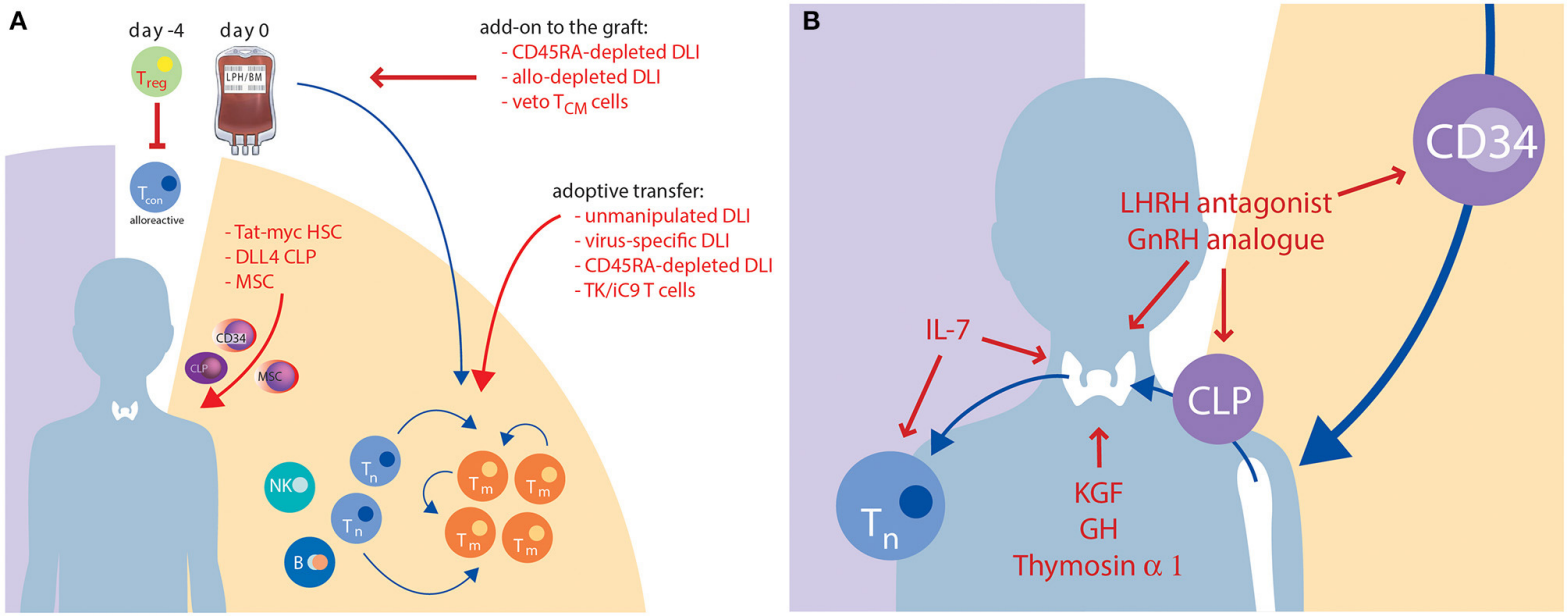
Current approaches to improve IR which are under clinical evaluation. This graph illustrates strategies with cellular therapies **(A)** or solubles factors **(B)** which are discussed above in sections Cellular Therapies, Soluble Factors, and Tissue Engineering. Red colour highlights the names, red arrows indicate the targets of the novel approaches. B, B cell; CLP, common lymphoid progenitor; DLL4, delta-like ligand 4; DLI, donor lymphocyte infusion; GH, growth hormone; GnRH, gonadotropine releasing hormone; HSC, haematopoietic stem cell; IL, interleukin; KGF, keratinocyte growth factor; LHRH, luteinizing hormone-releasing hormone; MSC, mesenchymal stem cell; NK, natural killer cell; T_CM_, central memory T cell; T_con_, conventional CD3^+^ T cell; T_m_, memory T cell; T_n_, naïve T cell; T_reg_, regulatory T cell; TK/iC9, thymidine kinase/inducible Caspase 9.

According to the two stages of T-cell reconstitution, efforts to improve IR in the clinical setting are based on two principles: (1) optimization of the peripheral (memory) T-cell compartment; and (2) enhancing of thymus-dependent (naïve) T-cell production. Cellular therapies are primarily based on modifications of graft composition aiming to optimise the peripheral T-cell compartment. Interventions including soluble factors and new concepts of tissue engineering may result in a better and/or faster thymic-dependent immunity. Findings from pre-clinical and clinical research in this area are summarised in Tables 2, 3, respectively, and described in more detail below (4, 14, 116, 158–194, 199–215, 220–222). Figure 2 graphically illustrates attempts to improve IR which are currently evaluated in clinical trials.

**Table 2:** Preclinical studies exploring soluble factors and cellular therapies to enhance T-cell function after HCT.

**Factor/method**	**Target**	**Selected recent references**
**Soluble factors**		
Interleukin-7*	Haematopoietic progenitor cells, thymocytes, peripheral T lymphocytes	(24, 158–161)
Interleukin-12	Thymocytes	(162, 163)
Interleukin-15	NK/NKT cells, CD8^+^ T cells	(164, 165)
Interleukin-21	Thymocytes, haematopoietic progenitor cells	(166)
Interleukin-22	Thymic epithelial cells	(167, 168)
FMS-like tyrosine kinase 3 (FLT3) ligand	Hematopoietic progenitor cells	(169–171)
Insulin-like growth factor 1	Thymic epithelial cells, myeloid cells	(172, 173)
Keratinocyte growth factor*	Thymic epithelial cells	(116, 174–177)
Receptor activator of NF-κB ligand (RANKL)	Thymic epithelial cells	(178), Montero-(179)
Stem cell factor	Thymocytes	(180)
Thymosin alpha 1*	Thymic epithelial cells, thymocytes	(181), (182)
Sex hormone ablation*	Thymic epithelial cells, thymocytes, haematopoietic progenitor cells	(183–186)
Growth hormone*	Thymic epithelial cells, thymocytes	(187)
**Cellular therapies**		
Precursor T cells (*ex vivo* generated by Notch-1 ligand delta-like-1 or Notch ligand delta-like-4 containing cocktails from HSC)*	Thymic epithelial cells, thymocytes	(188, 189)
Thymic epithelial cells (*ex vivo* generated by *Foxn1* containing cocktail from fibroblasts, embryonic stem cells or iPSCs)	Thymic epithelial cells, thymocytes	(190–192)
Mesenchymal stromal cells (*ex vivo* expanded)*	Haematopoietic progenitor cells, thymic epithelial cells, T cells	(193, 194)
Anti-Viral Central Memory CD8 Veto Cells*	Donor-specific host T cells, host leukemic cells, virally infected cells	(195, 196)
Regulatory T cells	Alloreactive conventional donor T cells	(197, 198)
Endothelial cells (*ex vivo* expanded)	Thymic epithelial cells	(199)
Injectable thymus organoids	Common lymphoid precursors, peripheral T cells	(200, 201)

Table adapted from Velardi et al. (6). ^*^Principles already investigated or being investigated in ongoing clinical studies. HSC, haemopoietic stem cell; iPSC, induced pluripotent stem cells.

**Table 3:** Clinical studies investigating approaches to enhance immune reconstitution after HCT and in patients with HIV.

**Factor/method**	**Description**	**Age group**	**Clinical trials in the HCT setting**	**References**
**Donor lymphocyte infusions (DLIs)**				
Unselected CD3^+^ T cells	Unseparated donor T cells	Paediatrics/adolescents/ adults	Treatment and prevention of relapse in malignant haematological diseases	(202)
Virus-specific CD3^+^ T cells	Enrichment of IFN-γ-secreting virus-specific T cells or by binding to viral peptide HLA tetramers after short stimulation *in vitro*		Pre-emptive treatment or therapy of infection by several viruses (EBV, CMV, adenovirus, HHV-6, BK polyomavirus)	(203–206)
DLIs armed with a suicide gene	Herpes simplex virus thymidine kinase suicide gene (HSV-TK cells); inducible caspase 9 suicide gene (iC9 T cells)		• Haploidentical HCT: HSV-TK cells (28 pts., Phase I/II) • iC9 T cells in malignant diseases, FHL, and XLP (15 pts., Phase I, active, not recruiting, NCT01494103), in malignant and non-malignant diseases (~200 paediatric pts., Phase II study, active, not recruiting, NCT02065869)	(207, 208)
CD45RA^+^-depleted CD3^+^ T cells	*In vitro* depletion of naïve T cells following MNC apheresis		• Allogeneic HCT, prophylactic and pre-emptive infusions (6 pts., pilot study) • High risk leukaemia, CD34-selected graft + CD45RA^+^-depleted DLI (35 pts., pilot study) • HLA-mismatched HCT in CID, chronic viral infections (5 paediatric pts., pilot study) • Treatment of CMV disease (1 pt.)	(14, 209–211)
Allo-depleted CD3^+^ T cells	*In vitro* depletion of allo-reactive T cells following MNC apheresis—*via* immunotoxins, reagents reacting with activation markers (CD25) or photodepletion		• Congenital haematological disorders (15 paediatric pts., Phase I/II) • Haploidentical HCT (15 pts., Phase I) • CD25/71 allo-depleted donor T cells vs. standard practise in adult malignancies (37 pts., randomised Phase I/II, completed 2020, NCT01827579) • Haploidentical HCT, allo-depleted vs. PTCy in adult malignancies (250 pts., randomised Phase III, active, not recruiting, NCT02999854)	Andre-(212–215)
Donor T_reg_	*Ex vivo* positively selected T_reg_ without expansion		• Haploidentical HCT, patients aged 18–65 years with high-risk acute leukaemias lacking a matched donor. • Haplo T_reg_ (2 × 10^6^/kg) day −4 combined with haplo T_con_ (1 × 10^6^/kg) day 0	(216, 217)
Anti-viral central memory CD8 veto cells	Central memory donor CD8^+^ T cells cultivated *ex vivo* under cytokine starvation in the presence of viral peptides.		• Haploidentical HCT after reduced intensity conditioning, Phase I/II, actively recruiting, NCT03622788. • Patients aged 12–75 years with haematologic malignancies, aplastic anaemia, severe immune deficiency or non-malignant bone marrow failure.	(218)
**Soluble factors**				
Interleukin-7	Target: HSPCs, thymocytes, peripheral T lymphocytes	Adults/adolescents	• T-cell-depleted HCT: expansion of effector memory cells, enhanced TCR diversity (8 pts. >15 years old, Phase I, published) • Non-HCT: treatment of HIV-1 pts. (Phase I; NCT00477321; NCT01190111, NCT01241643) • Idiopathic CD4 lymphocytopenia (21 pts.; Phase I/II, completed, NCT00839436, published)	(161, 219)
Keratinocyte growth factor (palifermin)	Target: thymic epithelial cells	Adults	• Allogeneic HCT in malignancies (6 adult pts.; randomised Phase I; completed, NCT01233921) • Autologous transplant in NHL (17 adult pts.; Phase I, completed; NCT03042585) • Haploidentical HCT in haematological malignancies (9 adult pts., randomised phase II, terminated NCT00593554) • Allogeneic HCT in malignancies (50 adult pts., phase I/II, recruiting; NCT02356159)	
Thymosin alpha 1	Target: thymocytes	Adults	HCT in malignant diseases (6 adult pts., randomised phase I/II study, completed, NCT00580450)	(181)
LHRH antagonist (degarelix)	Sex steroid ablation, target: thymic epithelial cells, bone marrow hematopoietic stem and progenitor cells, thymocyts	Paediatrics/adolescents/ adults	HCT in malignant diseases (76 paediatric and adult pts., randomised pilot study, completed; NCT01338987)	(185)
GnRH analogue (leuprolide)		Adults	T-cell-depleted HCT in malignant diseases: palifermin + leuprolide (82 adult pts., single-arm phase II, recruiting; NCT01746849)	
Growth Hormone	Target: thymic epithelial cells and thymocytes	Adults	• HIV patients (NCT00071240, NCT00287677, NCT00119769, NCT00050921) • No clinical trial in HCT setting	(220, 221)
**Stem cell engineering**				
TBX-1400 (Tat-MYC-transfusion protein)	Culture system with fusion proteins of the protein transduction domain of the HIV-1 transactivation protein (Tat) and MYC using HSC	Paediatrics	Allogeneic HCT in SCID pts. (8 paediatric pts., single arm, Phase I, not yet recruiting; NCT02860559)	(222)
Precursor T cells	Feeder-cell-free culture system based on the immobilised Notch ligand delta-like 4 using CD34^+^-selected HSC	Paediatrics	Haploidentical HCT in SCID pts. (12 paediatric pts., single arm, phase I/II, recruiting; NCT03879876)	(189)
MSCs	*Ex vivo* expanded mesenchymal stromal cells	Adults	Autologous transplantation in malignant lymphoma and multiple myeloma (pilot study)	(194)

*CID, combined immunodeficiency; CMV, cytomegalovirus; EBV, Epstein-Barr virus; FHL, Follicular Hodgkin lymphoma; GnRH, gonadotropin releasing hormone; HHV, human herpesvirus; HIV, human immunodeficiency virus; HCT, haematopoietic stem cell transplantation; HSPC, haematopoietic stem and progenitor cells; MSC, mesenchymal stroma cells; NHL, non, Hodgkin lymphoma; PTCy, post-transplant cyclophosphamide; SCID, severe combined immunodeficiency; TCR, T-cell receptor; T_reg_, regulatory T cell; XLP, X-linked lymphoproliferative disease*.

##### Cellular Therapies

Regarding cellular therapies, manipulation of the stem cell graft as well as use of DLIs are established modes to engineer T-cell immunity including anti-leukaemic effects (Figure 2A). The administration of unmanipulated donor lymphocytes is, however, complicated by a high risk of GvHD, which is even more relevant in an HLA-mismatched setting. Because of the adverse effects of GvHD on the thymus, unselected DLIs are not suitable to improve IR. Conversely, non-specific T-cell depletion of the graft, which is used particularly in HLA-mismatched HCT to avoid excessive GvHD, is complicated by delayed IR resulting in severe infectious complications and higher rates of graft rejection and relapse in patients with malignant diseases (223, 224). Advances in graft manipulation *in vivo* and *in vitro* aim to protect preferred T-cell subsets in order to maintain GvL and antiviral effects while reducing the risk of GvHD. The selective depletion of TCR-α/β lymphocytes spares the innate-like TCR-γ/δ population, thus possibly confering an improved anti-infective and antitumor response (74, 225). However, the anti-infective efficacy of TCR-γ/δ T cells is limited, and thymic-dependent IR is not improved by this procedure. Another approach using cyclophosphamide post HCT to prevent GvHD was pioneered by the Johns Hopkins group. This approach is widely used in adult patients with malignant and non-malignant diseases mainly but not exclusively in the HLA-mismatched setting (226, 227). A comparison of *in vitro* T-cell depleted allogeneic HCTs with post-transplant cyclophosphamide HCTs, including consideration of IR, is the topic of a separate review in this issue.

Several methods have been explored in the clinical setting to manipulate lymphocytes so that their anti-infectious activity is retained yet the risk of GvHD is reduced. The option of adoptive transfer of virus-specific T cells has already been mentioned above. Modern strategies allow rapid manufacturing of T cells against several viruses including EBV, CMV, adenovirus, HHV-6 and BK polyomavirus and are the subject of two previous reviews (203, 204). By magnetic enrichment of IFN-γ-secreting cells after short-term stimulation with viral peptide antigens, HLA-unrestricted viral-specific T cells can be produced within 1 day (205, 206). Virus-specific T cells from third-party donors are also in clinical use (228). They are usually readily available and are effective in mediating antiviral immunity without increasing the risk of GvHD (229). Another innovative approach is the generation of veto T cells with antiviral activity. This technique was developed by Reisner and colleagues and is based on the finding that T cells cultured with antigenic stimulation but under cytokine starvation are endowed with veto activity, i.e., the potential to eliminate host-vs.-graft-directed host T-cell clones, thereby facilitating donor engraftment even after reduced intensity conditioning (195) together with the preserved potential to kill host leukemic cells (196). If viral peptides are used for antigenic stimulation during *in vitro* culture of these cells, the veto T cells will confer graft facilitation together with improved antiviral IR in the early post-transplant phase (230). The first clinical results using the intended conditioning regimen (reduced intensity with post-transplant cyclophosphamide) followed by CD3/CD19-depleted haploidentical PBSCs were encouraging (231), and the utility of this approach in combination with veto T cell infusion is currently being investigated in a Phase I/II trial (ClinicalTrials.gov identifier: NCT03622788).

As outlined above (chapter 5.3) regulatory T cells are key regulators of alloreactivity and fast and sustained T_reg_ reconstitution is associated with lower incidences of GvHD and lower transplant-related mortality. Thus, several investigators have established approaches for adoptive transfer of these cells. Although T_reg_ products from third party cord blood units have been used as well, the majority of groups have relied on donor PMNCs as source of T_reg_. In a first feasibility trial, 28 adult patients grafted with CD34^+^ selected haploidentical PBSCs received on day −4 freshly isolated T_regs_ in a 2:1 ratio together with conventional T cells (216). Although only 2 out of 26 evaluable patients developed GvHD ≥ grade 2 and no SAEs were reported in association with the T_reg_ infusion, TRM was 50%, making efficacy assessment difficult. In a follow-up report of the same group, 43 adult patients with AML/ALL were transplanted using the same approach (217). After switching to a less toxic preparative regimen, TRM could be reduced to 21%. Albeit patients received a mean of 1.1 ± 0.6 × 10^6^ haploidentical CD3^+^/kg BW, GvHD incidences were comparable to a historical control group with fully T-cell depleted grafts. In order to increase transplantable cell numbers and to be compliant with current regulation, a GMP-compatible manufacturing process was developed, in which isolated T_reg_ were expanded with IL-2 and rapamycin (232). After 14 days of expansion, a 9.6 fold expansion was achieved with good suppressive function of the final T_reg_ product. This product now awaits testing in a tolerance induction protocol after haploidentical HCT.

A very intriguing yet easy to realise technique to reduce the alloreactivity of donor lymphocytes is the enrichment of memory T cells by CD45RA depletion. This technique and the first clinical results have been described in detail in the former sections of this review. An alternative and even more selective approach is selective allodepletion. Application of allodepleted T cells *in vitro* seems an attractive way to transfer antitumour and anti-infectious immunity from the donor to the recipient while avoiding the risk of GvHD. Reagents reacting with activation markers such as CD25, immunotoxins or a photodepletion procedure (using Kiadis Pharma technology) are methods to reduce alloreactive T cells for DLI (212–214). In two prospective randomised trials (Clinicaltrials.gov identifier: NCT02999854 and NCT01827579), such modified DLIs are currently being assessed vs. “standard” methods of haploidentical HCT, including the use of post-transplant cyclophosphamide (which was mentioned above).

Another approach for safer DLI administration involves T cells being armed with an inducible suicide gene. In a phase I-II, multicentre, non-randomised trial (ClinicalTrials.gov identifier: NCT00423124) in adult patients with high-risk haematological malignancies after haploidentical HCT, herpes-simplex thymidine kinase suicide gene expressing donor lymphocytes (HSV-TK) were infused after transplantation (207). Of the 28 patients receiving these HSV-TK cells after HCT, 22 obtained IR (i.e., CD3^+^ > 100/μl) at a median of 75 days (range 34–127 days) from transplantation and 23 days (range 13–42 days) from infusion. Ten patients developed aGVHD (grade I–IV) and one developed cGVHD, which was controlled by induction of the suicide gene. In another pilot study (Clinicaltrials.gov identifier: NCT01494103), 12 recipients of haploidentical HCT for different diseases including ALL, MDS, JMML, and HLH (medium age 10 years, range 2–50 years) were infused with increasing numbers of alloreplete haploidentical T cells expressing the inducible caspase 9 suicide gene (iC9-T cells) (208). All patients receiving >10^4^/kg of alloreplete iC9-T lymphocytes achieved rapid reconstitution of immune responses toward five major pathogenic viruses and concomitant control of active infections. By administration of a chemical inducer of dimerization (AP1903/rimiducid), 86–96% of circulating CD3^+^CD19^+^ T cells were eliminated within 30 min, with no recurrence of GvHD within 90 days (208). Another Phase II trial using this approach after haploidentical HCT with CD3^+^ TCRα/β-depleted grafts in about 250 paediatric patients with malignant and non-malignant diseases is ongoing in Italy and the UK (Clinicaltrials.gov identifier: NCT02065869). In an interim analysis, 10.9 and 2.1% of patients developed grade II–IV and grade III–IV aGvHD, respectively. 4.6% of patients [95% CoI: 1.3–7.8] developed cGvHD (233). Of 21 patients developing GvHD, 86% responded to rimiducid, with a median time to response of 2 days. Of initial responders, 77% were still in either complete (*n* = 8) or partial response (*n* = 6) at the time of interim analysis.

##### Soluble Factors

Although the above methods for graft manipulation and DLI engineering show promising results in host defence, they all carry the major disadvantage of expansion of memory-type T cells in the absence of a polyclonal naïve T-cell compartment. Since dysfunction of the thymus represents the limiting factor for full T-cell recovery, strategies to accelerate naïve, polyclonal, *de novo* T-cell reconstitution are warranted. Strategies proposed in recent years include the stimulation of T-cell development and expansion using (1) cytokines such as IL-7, IL-12 and IL-21; (2) the administration of cytokines alongside growth factors such as stem cell factor (also known as KIT ligand), keratinocyte growth factor (KGF encoded by the fibroblast growth factor 7 gene), IL-22 and FMS-like tyrosine kinase 3 ligand; and (3) the modulation of hormone levels by suppression of sex steroids or by administration of thymosin-α1. For a recent review see Velardi et al. (6). Some of these factors have recently been explored or are currently being explored in clinical trials in the context of HCT (Figure 2B and Table 3).

Members of the common gamma-chain cytokine family IL-7 and IL-15 are involved in homeostatic expansion of T cells in the peripheral blood (234). In mice and non-human primates, administration of IL-7 seems to have a positive effect on functional T-cell recovery after HCT, with a predominant effect on naïve CD8^+^ cells (24, 158, 159). However, this positive effect on thymus regeneration could not be confirmed in another animal study (160). In a phase I/II clinical trial treatment of 21 adult patients with idiopathic CD4^+^ lymphytopenia with recombinant IL-7 (without HCT) led to an increase in the number of circulating CD4^+^ and CD8^+^ T cells and tissue-resident CD3^+^ T cells in the gut mucosa and bone marrow; however, enhanced thymospoiesis, measured by TRECs, was only observed in the youngest patients, aged 23 and 34 years (NCT00839436) (219). In a phase I trial, 12 patients more than 15 years of age were treated with recombinant IL-7 after TCD allo-HCT from an 8 of 8 HLA-matched donor for treatment of non-lymphoid haematologic malignancy. After a short course of IL-7, a quantitative increase of CD4^+^ and CD8^+^ effector memory T cells as well as an increase in mitogen-reactive T cells was found (NCT00684008) (161). However, there was only a limited effect on thymic output in this study as shown by minimal changes in the number of recent thymic emigrants and the levels of TRECs. An extended duration of IL-7 administration may have a greater effect on thymic function particularly in younger patients. IL-7 is currently under investigation in multiple randomised clinical trials for oncologic and infectious disorders (including human immunodeficiency virus and severe acute respiratory syndrome coronavirus 2 infection), but there are no further studies in the allogeneic HCT setting to our knowledge. Taken together, the direct impact of IL-7 on the human thymus is still unclear, but most of the effects on T-cell IR after IL-7 treatment seem to be primarily related to the expansion of peripheral T cell subsets and to the improvement of T-cell functionality. A possible impact on thymic function seems to be restricted to younger patients with more residual thymic capacity.

IL-15 has been shown to increase the number of CD8^+^ T cells and NK cells after transplantation in mice (164). Similarly to IL-7, IL-15 can improve lymphocyte reconstitution after T-cell-depleted HCT, but it can also worsen GvHD, which limits its use in HCT (235). For a review see Moutuou et al. (236).

Factors that stimulate the thymic niche and increase the output of recent thymic emigrants, including KGF and the luteinizing-hormone–releasing hormone (LHRH) agonist leuprolide have been identified in mouse models (237, 238). Two trials are evaluating the effects of leuprolide and the LHRH antagonist degarelix on IR following HCT (ClinicalTrials.gov identifiers: NCT01746849 and NCT01338987), but results have not yet been reported.

Human recombinant KGF (palifermin) is approved by the US Food and Drug Administration for the prevention of mucositis in patients receiving high-dose chemotherapy including conditioning for HCT. In several mouse models KGF enhanced recovery of thymic cellularity and peripheral T cell numbers after HCT, reversed thymic involution and restored thympopoiesis (116, 174). Several trials are exploring its effects on T-cell reconstitution, but results have not been reported so far (ClinicalTrials.gov identifiers: NCT01233921, NCT03042585, NCT02356159, and NCT00593554).

Thymosin-alpha1 is a low molecular weight peptide produced by thymus epithelial cells, which can increase thymocyte maturation and boost T cell function as shown in several preclinical studies. In a phase I/II clinical trial the safety and efficacy of Thymosin-alpha1 was evaluated in 6 adult recipients of haploidentical HCTs for haematologic malignancies (ClinicalTrials.gov identifiers: NCT00580450) (181). An increase of peripheral T-cell numbers, an earlier appearance of pathogen-specific T cell responses as well as a significant improvement in phagocytosis and dendritic cell function was observed (181). However, to the best of our knowledge, there are no further trials ongoing exploring Thymosin-alpha 1 in the HCT setting.

##### Tissue Engineering

*De novo* T-cell generation is dependent on the continuous seeding of the thymus by T-lymphoid precursors. These T-lymphoid precursors must differentiate from donor-derived haematopoietic stem cells in the recipient bone marrow before they can home to the thymus *via* the peripheral blood. Since this process is compromised after HCT by damage to the thymus caused by total body irradiation, chemotherapy, infections and predominantly GvHD prophylaxis and treatment, it may take up to 2 years before T-cell neogenesis is re-established (3, 239, 240). Adoptive transfer of *in vitro* generated human T-lymphoid precursors is therefore a promising approach to shortcut this pathway by targeted injection of T-lymphoid progenitors.

An US group has developed a novel approach to expand a cytokine-dependent, haematopoietic progenitor cell population *ex vivo* by culturing primary haematopoietic stem and progenitor cells with fusion proteins comprising the transduction domain of the HIV-1 transactivation (Tat) protein and either MYC or B-cell lymphoma 2 (BCL-2) proteins (222). In both humans and mice, the *ex vivo* expanded cells gave rise to a self-renewing cell population following initial transplantation *in vivo*; serial transplantations of this cell population were able to support haematopoiesis. Based on these laboratory studies, a clinical trial has been initiated in Israel to assess the application of TBX-1400 in patients with severe combined immunodeficiency (human donor haematopoietic stem and progenitor cells that have been treated *ex vivo* with the protein transduction domain of the Tat fused to MYC, ClinicalTrials.gov identifier: NCT02860559).

Several other groups have developed systems to pre-differentiate T-lymphoid progenitors out of CD34^+^ haematopoietic stem cells, e.g., by using the canonical Notch ligand Delta-like (DL)-1, or more recently a French group using immobilised DL-4 (241). These techniques allow the *in vitro* generation of large amounts of T-cell progenitor cells with high T-lymphopoietic potential. When co-transplanted together with CD34^+^ haematopoietic stem cells, these committed precursors led to rapid T-cell engraftment within 28 days in a humanised mouse model (242). This protocol was improved in recent years to expand CD34^+^ cells from granulocyte colony-stimulating factor (G-CSF)-mobilised peripheral blood as well (189). After 7 days of *in vitro* culture, these cells expressed T-lineage-related, thymus homing and crosstalk genes as well as markers of early lymphoid commitment but do not show any TCR rearrangement. Remarkably, in a humanised mouse model, thymic engraftment occurred 4 weeks after intrahepatic injection of such precursors in comparison to 12 weeks after injection of uncultured, CD34^+^-selected haematopoietic progenitor cells (189, 243). Thus, T-lymphoid progenitors seems to allow thymic engraftment just 4 weeks after transfer, a result which has to be confirmed in the human setting. Since the injected precursors do not harbour any TCR rearrangement, they should allow the generation of a polyclonal and self-tolerant T-cell repertoire without increasing the risk of GvHD. A Phase I/II clinical trial was initiated recently to evaluate the safety and efficacy of human T lymphoid progenitor transfusion after haploidentical HCT in patients with severe combined immunodeficiency (Clinical trial identifier: NCT03879876).

In the case of an entirely a functional thymus, transplantation of postnatal allogeneic thymic tissue may be another option. This procedure improved thymic output in patients with complete DiGeorge syndrome (244, 245). Although this approach has not been tested so far after HCT, it has been investigated in patients with acquired immunodeficiency syndrome (246). However, in these patients, residual host T cells led to a high rate of thymic tissue rejection. Therefore, complete T-cell depletion prior to thymus transplantation is a potential requirement if this approach is to be trialled post HCT.

In summary, several strategies to accelerate recovery of T-cell immunity after allogeneic HCT are currently under clinical evaluation. Patients with prolonged immune dysfunction caused by chemotherapy, irradiation, infection and GvHD may benefit from a multifactorial approach. The combined use of optimised graft composition, soluble factors (IL-7, KGF, Thymosin-alpha-1), T-lymphoid progenitors or, in case of complete thymic involution, thymus tissue transplantation may be able to accelerate restoration of the T-cell compartment.

## Final Remarks

Studies about the reconstitution kinetics of different cellular subsets after HCT have revealed important insights about the basic principles of this treatment. They helped us to understand the artificial immune ontology after HCT as well as the pathophysiology of GvHD, viral reactivation and other transplant-related complications. By continuous efforts to dissect the phenomenon of alloreactivity, IR studies have opened the door to understand the GvL effect, at least in part. In recent years, research on IR has evolved from merely descriptive studies into a highly dynamic and innovative field which actively shapes the future design of HCT. Novel insights have fostered the continuous evolution of T-cell-depletion techniques to a level by which HCTs employing this method now yield comparable results to T-replete HCTs. Clinical trials over the coming years will show whether adoptive transfer of memory DLI, veto T_CM_ cells or selective allodepletion approaches will give superior results. Strategies of restoring thymic cellularity by soluble factors, targeted influx of committed lymphoid progenitors or tissue engineering not only intend to lift IR kinetics of adult patients to that of an infant but will beyond that impact on ageing research since thymic involution is considered a major contributor of immune senescence.

Future studies on IR will aim to develop more precise prediction models for complications such as GvHD, viral disease or relapse. To this end, multifactorial models of IR will have to take the complex interactions around HCT into account and include not only lymphocyte subset numbers but also other factors such as graft type, graft manipulation, HLA disparity and minor histocompatibility differences. The first examples of such multidimensional IR analyses have already been published (71, 82, 132). Control over IR with targeted interventions in a timely orchestrated fashion will help to reduce transplant-related morbidity and mortality and improve GvHD-free, relapse-free survival.

## Author Contributions

AY, AS, AL, SN, and ME contributed specific chapters to this review article. All authors contributed to the article and approved the submitted version.

## Funding

This study received funding from the St. Anna Children's Cancer Research Institute, Vienna, Austria. The funders were not involved in the study design, collection, analysis, interpretation of data, the writing of this article, or the decision to submit it for publication.

## Conflict of Interest

The authors declare that the research was conducted in the absence of any commercial or financial relationships that could be construed as a potential conflict of interest.

## Publisher's Note

All claims expressed in this article are solely those of the authors and do not necessarily represent those of their affiliated organizations, or those of the publisher, the editors and the reviewers. Any product that may be evaluated in this article, or claim that may be made by its manufacturer, is not guaranteed or endorsed by the publisher.
